# A Case of Morganella morganii-induced Fetal Demise

**DOI:** 10.7759/cureus.7169

**Published:** 2020-03-03

**Authors:** Sofanit A Dessie, Varun Dobariya, Davinder Singh, Peimei He

**Affiliations:** 1 Internal Medicine, Marshall University, Joan C. Edwards School of Medicine, Huntington, USA; 2 Internal Medicine, Marshall University, Huntington, USA

**Keywords:** morganella morganii, fetal demise, premature prolonged rupture of membranes

## Abstract

Morganella morganii is a rare opportunistic pathogen that is known to cause urinary tract and intra-abdominal infections. Per our literature review, there are few case reports of neonatal sepsis associated with this organism but to our knowledge, there are no case reports of Morganella morganii causing fetal demise in published literature in the Englishlanguage. In this case report, we present a case of a 34-year-old pregnant woman who had a hospital course complicated by Morganella morganii, which eventually led to stillbirth.

## Introduction

Morganella morganii is a facultative, anaerobic, gram-negative rod belonging to the Enterobacteriaceae family. The bacteria, which is usually found as normal flora in the human gut, is known to cause urinary tract infections, as well as intra-abdominal infections, especially of the hepatobiliary tract [1-3]. Old age, hospitalization, surgery, antibiotic use, and concomitant bacteremia have been reported as the most common risk factors [4]. In regard to the pediatric population, Morganella morganii has been a reported cause of neonatal sepsis, meningitis, and chorioamnionitis but rarely implicated in fetal demise [5]. Herein, we present a case of fetal death in a 34-year-old female who presented with premature prolonged rupture of membrane (PPROM).

## Case presentation

A 34-year-old gravida 5 woman in her 27.5 week of pregnancy presented to our institution as a transfer from an outside hospital for PPROM. Her prenatal course was complicated by a short cervix for which she had cerclage placement six weeks before her presentation. Her past medical history was significant only for hypertension. Obstetric history included two uncomplicated, spontaneous vaginal deliveries and two elective abortions. Her social history is significant only for occasional smoking of marijuana but no intravenous drug, alcohol, or tobacco use. Intravaginal progesterone gel and prenatal vitamins were her only home medications. Vital signs were stable at presentation, including blood pressure of 110/76 mmHg, heart rate of 90 beats per minute, respiratory rate of 18 breaths per minute, temperature of 36.8°C, and oxygen saturation of 97% on room air. Physical examination was evident for a grossly ruptured amniotic membrane with non-foul smelling clear amniotic fluid, one cm dilated cervix, and cerclage in place. There was no contraction detected by the tocometer. Laboratory work was unremarkable, including white blood cell count (WBC) of 9.7 x 109/L and hemoglobin of 13.5 g/dL. Urinalysis, urine drug screening, human immunodeficiency virus antibody, chlamydia, and gonorrheal polymerase chain reactions were all negative. She was started on prophylactic intravenous (IV) 2000 mg ampicillin every six hours, and she received it for 48 hours. On day two, the cerclage was removed and she was continuously monitored. On day three, her WBC increased to 11.4 x 109/L, but she remained afebrile, the cervix remained unchanged, and fetal heart rates were reassuring. Trichomonas and bacterial vaginosis were discovered on a wet mount and she received metronidazole. Urine culture grew Morganella morganii and nitrofurantoin was started. The next day, she developed a fever of 39.5°C. WBC increased to 17.1 x 109/L and lactic acid was elevated at 4 mmol/L. An emergency caesarian section was done on the same day for non-reassuring fetal status with frequent fetal decelerations and fetal bradycardia. Unfortunately, the fetus was delivered without a heartbeat despite immediate attempts of cardiopulmonary resuscitation. There was no foul-smelling or purulent lochia in the postoperative period. Blood and placental cultures came back suggestive of multidrug-resistant Morganella morganii but sensitive to piperacillin-tazobactam and meropenem. She was started with intravenous (IV) piperacillin-tazobactam and the sepsis resolved subsequently.

## Discussion

A literature search shows few case reports and studies of early-onset neonatal sepsis caused by Morganella morganii. One literature review for neonatal sepsis points out that 10 out of 11 neonates had early-onset sepsis and nine of them were preterm. Maternal chorioamnionitis was the most described antenatal risk associated. The risk factors for our patient include cerclage placement, PPROM, and treatment with prophylactic ampicillin, which has been reported to increase the risk of infection with ampicillin-resistant organisms, including Morganella morganii [6-7]. Maternal urinary tract infection with this organism associated with PPROM should prompt clinicians to treat with appropriate antibiotics and consider the delivery of the fetus at the earliest sign of intrauterine stress. Maternal sepsis with Morganella morganii should be managed closely to prevent intrapartum fetal demise. Culture and sensitivity should guide the choice of antibiotic selection. Morganella morganii is usually resistant to many beta-lactam antibiotics so third-generation cephalosporine can be used alone or in combination with gentamicin for 10-14 days in uncomplicated cases, but culture and sensitivity should guide the choice of antibiotic selection [7].

## Conclusions

Morganella morganii is an opportunistic pathogen that rarely causes neonatal bacteremia and sepsis. Our case describes a unique event where this organism led to fetal demise and, hence, we recommend clinicians to have a high index of suspicion for this association to prevent a fatal outcome.
